# Sunflower Receptacle Extract Alleviates Acute Gouty Arthritis via HMGB1-TLR4-NLRP3 Inflammatory Pathway

**DOI:** 10.3390/nu18193290

**Published:** 2026-10-07

**Authors:** Zhiman Li, Zhilong Li, Xiaohui Huo, Xinyuan Wang, Yinshi Sun

**Affiliations:** 1 College of Chinese Medicinal Materials, Jilin Agricultural University, Changchun 130118, China; 2 Institute of Special Animal and Plant Sciences, Chinese Academy of Agricultural Sciences, Changchun 130112, China; 13234437598@163.com (Z.L.);; 3 Dong-E Ejiao Health Industry Technology Co., Ltd., Liaocheng 252201, China

**Keywords:** sunflower receptacle extract, acute gouty arthritis, HMGB-1, TLR4, NLRP3

## Abstract

**Background/Objectives:** Acute gouty arthritis (AGA) is an auto-inflammatory disorder characterized by dysregulated innate immune activation and overactive NOD-like receptor family pyrin domain-containing 3 (NLRP3)/Toll-like receptor 4 (TLR4) inflammatory signaling. While adverse effects limit current therapies, the mechanism by which sunflower receptacle extract (SPE) alleviates gout remains unclear. **Methods:** This study investigated the anti-inflammatory mechanism of SPE in a male SPF-grade SD rat model of AGA induced by intra-articular monosodium urate crystals. Rats were orally administered SPE or colchicine, and ankle joint swelling, synovial histopathology, serum levels of tumor necrosis factor-α (TNF-α), interleukin-1β(IL-1β), and interleukin-6 (IL-6), and synovial protein expression were assessed. Western blotting was used to assess the expression of the high-mobility group box 1 (HMGB-1), TLR4, and mitogen-activated protein kinase (MAPK) signaling pathways, as well as the activation of NLRP3. **Results:** Compared to the AGA group, treatment with colchicine and SPE significantly reduced ankle joint swelling, improved inflammatory infiltration in synovial tissue, and lowered serum levels of inflammatory factors TNF-α, IL-1β, and IL-6 (*p* < 0.05, *p* < 0.01). They also markedly decreased the expression of HMGB-1 and proteins in the TLR4, nuclear factor-ĸB (NF-ĸB), and MAPK pathways in joint synovial tissue (*p* < 0.05, *p* < 0.01, *p* < 0.001). **Conclusions:** SPE markedly reduced joint swelling and synovial inflammatory infiltration, lowered serum TNF-α, IL-1β, and IL-6 levels, and decreased synovial expression of HMGB-1, TLR4, MyD88, TRAF6, and NF-ĸB. Moreover, SPE suppressed NLRP3 inflammasome assembly and MAPK pathway activation. These results indicate that SPE mitigates AGA by inhibiting the HMGB-1-TLR4-NLRP3 inflammatory axis.

## 1. Introduction

Gout is an inflammatory joint disease caused by the formation of monosodium urate crystals. The main symptoms are joint redness, swelling, burning, and pain [1,2]. Hyperuricemia, due to purine metabolism and decreased uric acid excretion, is the main risk factor for MSU crystal formation [3]. Gout is usually characterized by frequent acute arthritis episodes, with symptoms usually resolving spontaneously within 7–10 days, and patients are asymptomatic between attacks [4]. In recent years, the global prevalence of gout has been increasing worldwide. The worldwide surge in gout cases underscores its identity as a metabolic disorder rooted in impaired purine metabolism and sustained hyperuricemia [5,6]. Although common drugs like febuxostat, benzbromarone, and colchicine can control gout to some extent, long-term use can lead to liver and kidney damage, gastrointestinal problems, and other adverse effects [7,8]. Therefore, finding safe and effective natural products with minimal adverse effects and understanding their anti-gout mechanisms is theoretically crucial and clinically useful for developing new strategies for prevention and treatment [9].

Sunflower (*Helianthus annuus* L.) is an annual herbaceous plant from the family Asteraceae, which was estimated to cover 4.8 million hectares globally in 2023, yielding 10 million tons of seeds. It is the third-largest imported oil crop in Europe [10]. Sunflower receptacle contains many fatty acids, flavonoids, amino acids, organic acids, polysaccharides, and volatile oils. As documented in the *Shennong Bencao Jing*, sunflower receptacle has traditionally been used for its anti-inflammatory and analgesic properties [11]. Sunflower receptacle is often used as feed or waste, leading to resource waste. Current research into its medicinal properties and active ingredients offers new avenues for the high-value utilization of this by-product [12]. Modern pharmacological studies have demonstrated that sunflower receptacle extract can regulate oxidative stress, modulate anti-inflammatory cytokines, and inhibit xanthine oxidase (XOD) activity, thereby lowering uric acid levels to exert an anti-gout effect [13]. Sunflower receptacle enzymatic hydrolysates can inhibit the activities of xanthine oxidase (XOD), adenosine deaminase (ADA), and urate transporter1 (URAT1), and improve renal function abnormalities mediated by hyperuricemia [14]. Sunflower receptacle water extract can decrease uric acid levels by regulating URAT1 and ATP-binding cassette transporter G2 (ABCG2) expression and inhibiting glucose transporter9 (GLUT9). It also reduces renal inflammation and repairs renal damage via the Cyclooxygenase-2 (COX-2)/Prostaglandin E_2_(PGE_2_) pathway [15]. These studies highlight the potential value of sunflower receptacle, but they focus solely on uric acid metabolism and inflammation in acute gouty arthritis. However, the core link of synovial inflammation in acute gouty arthritis has not been verified. Thus, this study targets the TLR4 signaling pathway mediated by HMGB-1 as the core target, based on network pharmacology research. Using animal models, this study aims to detect the expression of key molecules of HMGB-1, TLR4, and downstream NF-ĸB and MAPK pathways in rat synovial tissue to elucidate the core molecular targets and mechanisms by which SPE interferes with acute gouty arthritis.

## 2. Materials and Methods

### 2.1. Reagents

Colchicine and monosodium urate were obtained from Sigma-Aldrich (Shanghai) Trading Co., Ltd., Shanghai, China. An IL-6 rat ELISA kit, TNF-α rat ELISA kit, and IL-1β rat ELISA kit were obtained from Jiangsu Feiya Biotechnology Co., Ltd., Nantong, China. Radio-immunoprecipitation assay (RIPA) lysis buffer was acquired from Bio-World Company, Franklin, OH, USA, while a Bicinchoninic acid (BCA) kit was purchased from Beyotime Biotechnology Co., Ltd., Shanghai, China. Anti-glyceraldehyde-3-phosphate dehydrogenase (GAPDH) and mouse anti-IgG antibodies were obtained from Abcam, Waltham, MA, USA. Anti-NLRP3, anti-TLR4, anti-interleukin-1 receptor-associated kinase 1 (IRAK1), and anti-tumor necrosis factor receptor-associated factor 6 (TRAF6) antibodies were acquired from Santa Cruz Biotechnology Company, Dallas, TX, USA. Additionally, anti-caspase-1, anti-apoptosis-associated speck-like protein containing a CARD (ASC), anti-NLRP3, anti-Myeloid differentiation primary response 88 (MyD88), and anti-TLR4 antibodies were purchased from Beijing Biosen Biotechnology Co., Ltd., Beijing, China.

### 2.2. Preparation of Sunflower Receptacle Extract

Sunflower receptacles from Baicheng, Baotou, and Altay were used as raw materials. After ultrafine grinding to obtain uniform powder, 1 kg was measured. Then, 10 L of deionized water was added with a material-to-liquid ratio of 1:10 (g:mL). The sample bag was put in a boiling water bath for 2 h at a constant temperature, stirring every 15 min. After extraction, the supernatants were filtered and collected. Next, 10 L of deionized water was added to the filter residue, and the boiling water bath extraction was repeated under the same conditions. The second supernatant was mixed thoroughly, dried, and ground into powder. The extraction yields of the three extracts from Baicheng, Baotou, and Altay were 21.69%, 27.30%, and 28.72%, respectively.

### 2.3. Experimental Animals

Fifty-four healthy male SPF-grade SD rats (8-week-old, body weight 180–220 g) were obtained from Liaoning Changsheng Biotechnology Co., Ltd. [animal production license: SCXK (Liaoning) 2022-0001]. The experiment was started after 7 days of acclimatization. Rats were housed in IVC cages. Animal health status was monitored daily. No adverse events or deaths occurred during the experiment. No formal humane endpoints were predefined in this study. The rats were housed at 22 ± 2 °C, relative humidity 55% ± 15%, and 12 h light cycle. The experiment was approved by the Experimental Animal Management and Ethics Committee of the Institute of Animals and Plants of the Chinese Academy of Agriculture (NO.ISAPSAEC-2020-021-01).

### 2.4. Method

#### 2.4.1. Protein–Protein Interaction (PPI) and Kyoto Encyclopedia of Genes and Genomes (KEGG) Analyses

Active ingredients were first curated from the China National Knowledge Infrastructure CNKI (used solely as a bibliographic resource) and the phytochemical literature (up to June 2025), then filtered using the Traditional Chinese Medicine Systems Pharmacology Database and Analysis Platform (TCMSP, v2.3) and SwissADME for oral bioavailability (OB ≥ 30%) and drug-likeness (DL ≥ 0.18). Targets were predicted using Swiss Target Prediction (probability > 0.5), standardized via UniProt, and intersected with gout-related targets from GeneCards, OMIM, DrugBank, and DisGeNET. A drug–ingredient–target network was constructed using Cytoscape 3.7.2, and protein–protein interactions were analyzed via STRING (v12.0, Homo sapiens, confidence ≥ 0.7); hub genes were identified by degree, betweenness, and closeness centrality (top 30%). Gene Ontology (GO) and KEGG enrichment analyses were performed using Database for Annotation, Visualization and Integrated Discovery (DAVID, v6.8), with *p* < 0.05 considered statistically significant.

#### 2.4.2. Establishment of Acute Gouty Arthritis Model

Rats were assigned to different experimental groups using a random-number table. No additional dedicated procedures were applied to control confounding factors beyond standard laboratory breeding conditions. SD rats were randomly divided into a normal group (NC); acute gouty arthritis model group (AGA); colchicine-positive group (COL) 0.3 mg/kg; Baicheng sunflower receptacle low-dose group (BC-SPE-L) 0.5 g/kg; Baicheng sunflower receptacle high-dose group (BC-SPE-H) 1.0 g/kg; Baotou sunflower receptacle low-dose group (BT-SPE-L) 0.5 g/kg; Baotou sunflower receptacle high-dose group (BT-SPE-H) 1.0 g/kg; Altay sunflower receptacle low-dose (AT-SPE-L) 0.5 g/kg; and Altay sunflower receptacle high-dose (AT-SPE-H) 1.0 g/kg groups; with 6 rats in each group (*n* = 6 per group). The sample size was determined based on previously published related studies without formal a priori power calculation. Rats in each administration group received gavage for 7 days. On the 5th day, the AGA model was established, and 0.3 mL of MSU crystals (30 g/L) was injected into the left ankle joint cavity of each rat. Using MSU injection as the starting point, the ankle joint circumference (cm) of experimental rats was measured at 0 h before modeling and 4, 12, 24, and 36 h after modeling, and orbital blood was taken.

#### 2.4.3. Determination of Swelling Rate of Rat Ankle Joints

The ankle joint circumference (cm) of the rats was measured 5 times before modeling, and at 4 h, 12 h, 24 h, and 36 h after modeling. The swelling rate of the ankle joint was determined as follows: swelling rate = [ankle joint circumference after modeling − ankle joint circumference before modeling)/ankle joint circumference before modeling] × 100%.

#### 2.4.4. H&E Staining

The synovial tissue from the rat ankle joint was fixed in formalin, decalcified with an EDTA solution, and embedded in paraffin. It was then sectioned, stained with H&E, dehydrated, and sealed with transparent neutral gum. Pathological changes in the synovial tissue were subsequently observed under a microscope.

#### 2.4.5. Quantification of Inflammatory Factors

Taking the time of MSU injection as the starting point, orbital blood samples of rats were collected before modeling and at 4 h, 12 h, 24 h, and 36 h after modeling. The contents of inflammatory factors IL-6, IL-1β, and TNF-α in the serum of rats at different time periods were determined. The operation was strictly performed in accordance with the accompanying instructions of the kit.

#### 2.4.6. Western Blotting Analysis

The synovial tissue of the rat ankle joint was taken, and the total tissue protein was extracted using RIPA lysis buffer with phenylmethanesulfonyl fluoride (PMSF). The supernatant was collected after centrifugation, and protein concentration was measured using the BCA method as follows: Take an equal amount of protein sample, separate it using 8–12% separation gel SDS-PAGE electrophoresis, and transfer it to a polyvinylidene difluoride (PVDF) membrane. Use 5% skim milk blocking solution for 2 h at room temperature, wash 4 times with phosphate-buffered saline with Tween-20 (PBST), add primary antibody, and incubate overnight at 4 °C. After washing 3 times with PBST, add the secondary antibody and incubate at room temperature for 2 h at room temperature. After enhanced chemiluminescence (ECL) color development, images were collected in a versatile imaging system. ImageJ software (version: 1.x) was used to analyze the gray value of Western blot bands. The next steps were to normalize using GAPDH as an internal reference, calculate relative expression of the target protein, and compare differences between groups.

#### 2.4.7. Immunofluorescence Staining

Rat ankle joint synovial tissue sections were waxed, put in citrate buffer, and incubated for 20 min for antigen retrieval. They were then washed 3 times with phosphate-buffered saline tween (PBS) for 5 min, incubated with 3% H_2_O_2_ for 10 min, and washed 3 times with PBS for 2 min, then primary antibodies were added and incubated overnight at 4 °C. The next steps are as follows: Return to room temperature for 30 min. Add secondary antibody and incubate at room temperature for 1 h in the dark. After washing 3 times with PBS for 5 min, counterstained cells with DAPI staining solution to label nuclear DNA were added. It was then incubated at room temperature for 10 min in the dark. Fluorescence microscopy was performed.

#### 2.4.8. Statistical Analysis

All data were presented as mean ± SD. The statistical analysis of the data was processed using GraphPad Prism 8.0.1 software. For ankle swelling rate and serum inflammatory marker levels, intergroup comparisons were conducted stratified by each individual evaluation time point, with the primary endpoint being differences across treatment groups at each predefined time point. One-way ANOVA followed by Tukey’s post hoc test was used to compare data groups, and *p* < 0.05 indicated significant differences.

## 3. Results

### 3.1. Analysis of Network Pharmacology Results

**Figure 1:**
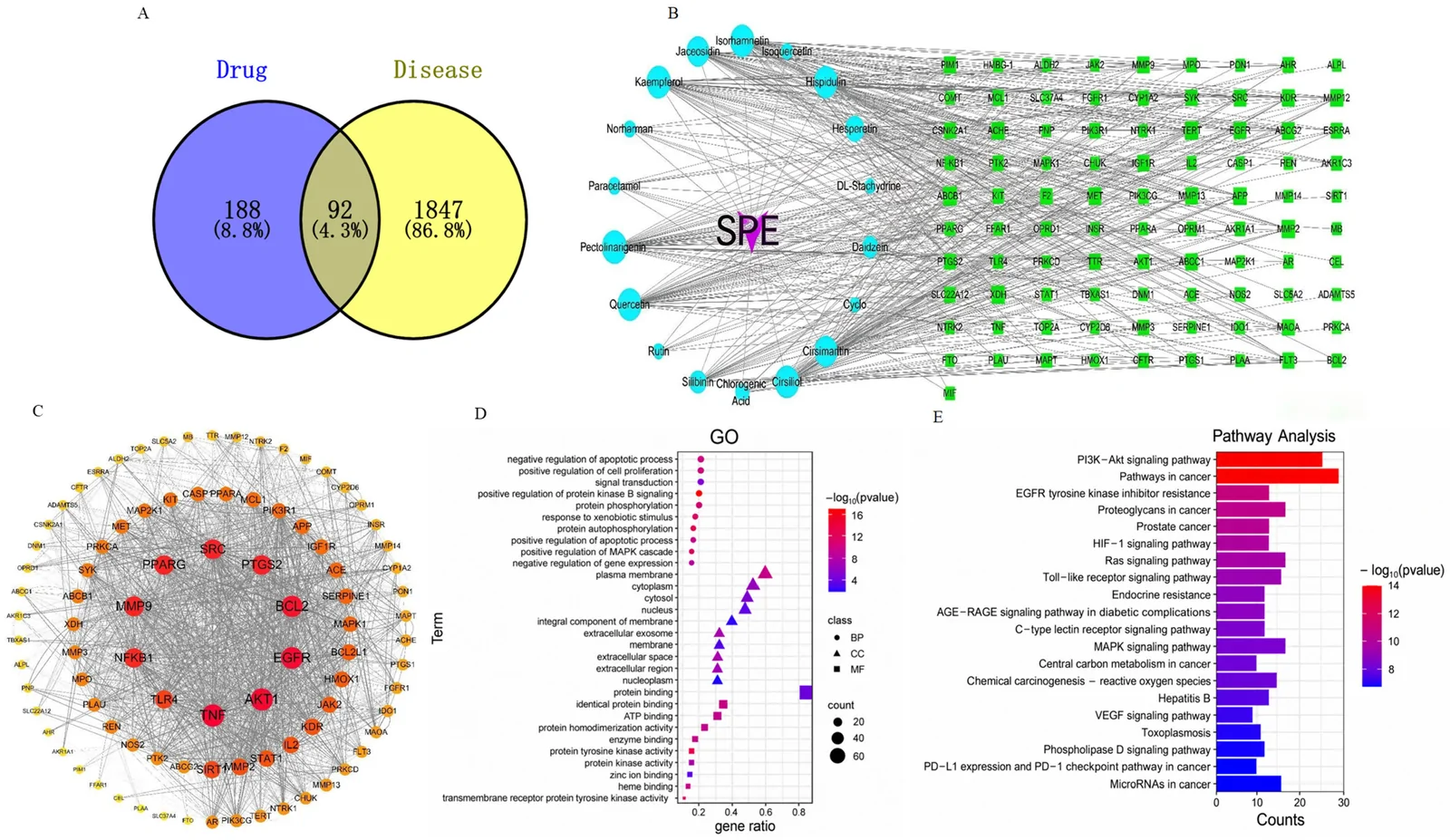
Network pharmacology analysis of the anti-gout mechanism of the sunflower receptacle. (**A**) Venn diagram of overlap between SPE targets and gout targets. (**B**) Drug–ingredients–target network. (**C**) Comparison between drug core target and disease target network plot. (**D**) Comparison of the GO functional enrichment plot. (**E**) Comparison with the KEGG pathway enrichment plot.

The Venn diagram results showed that combining sunflower receptacle target genes with gout targets 92 common target genes, which were active targets for gout drugs (Figure 1A). Among 18 potentially active components in sunflower receptacle, AGA disease targets are classical inflammatory proteins like HMGB-1 and TLR4 (Figure 1B). PPI network analysis showed that genes like TNF, proto-oncogene tyrosine-protein kinase Src (Src)SRC, matrix metalloproteinase9 (MMP9), peroxisome proliferator-activated receptor G (PPARG), NF-κB and TLR4 were central targets in the network, suggesting they may be targets for gout drug treatment (Figure 1C). GO analysis showed that biological processes notably enrich items were related to programmed cell death, mitosis regulation, and intracellular signaling pathways. At the cell component level, major items were concentrated in plasma membrane structures and cytoplasmic regions. Protein interactions and enzyme activity regulation were important (Figure 1D). The KEGG bubble diagram showed that TLR and MAPK signaling pathways are closely related to physiological processes such as cell death, proliferation, antioxidant activity, and inflammation (Figure 1E).

### 3.2. Effect of SPE on Ankle Joint of Rats with Acute Gouty Arthritis

**Figure 2:**
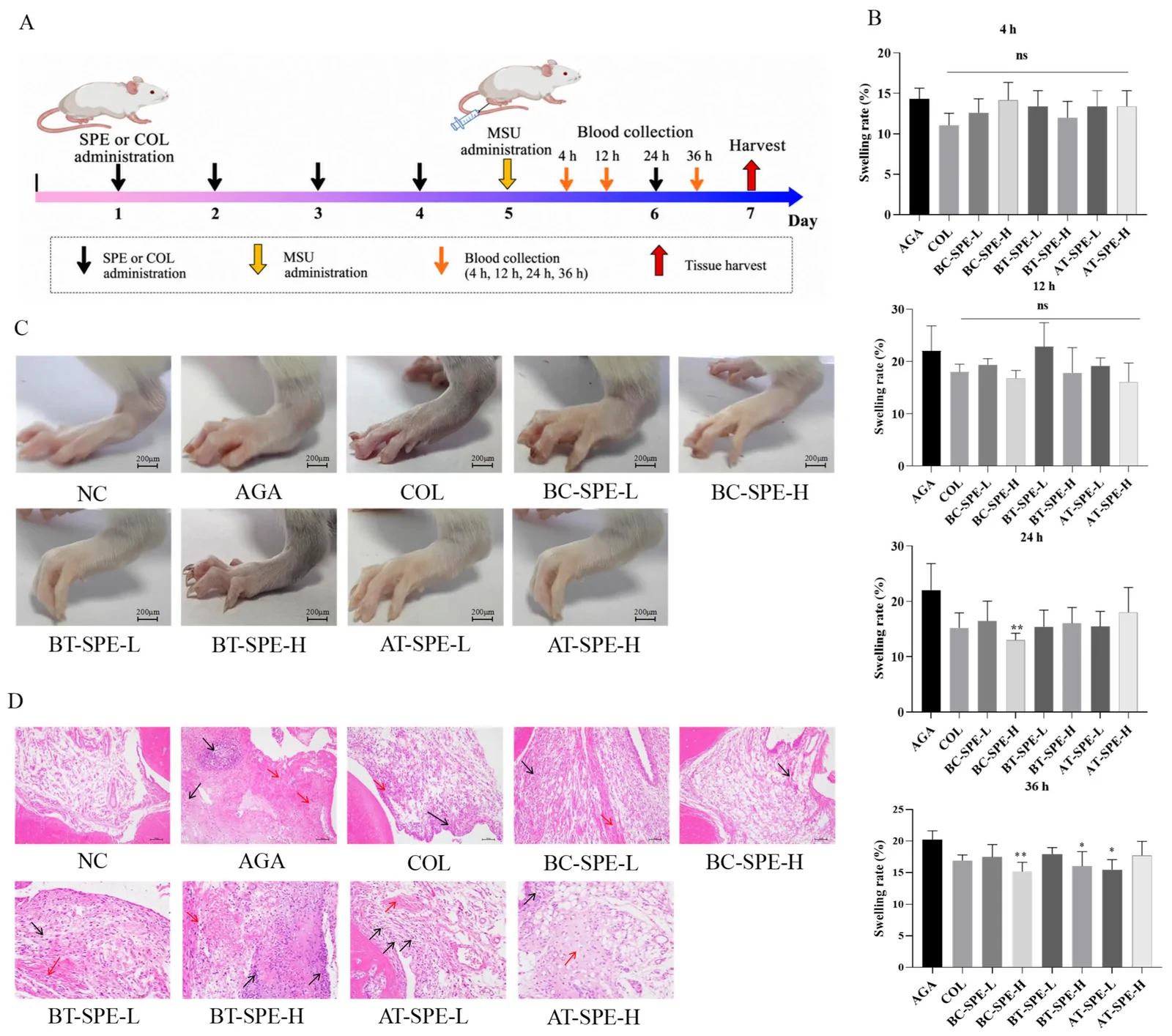
Effect of SPE on the ankle joint of rats with AGA. (**A**) Chart depcting the administration and modeling test process. (**B**) Swelling rate of the rat ankle joint at different times. (**C**) Effect on the morphology of the rat ankle joint. (**D**) Effect on histopathological changes in the synovial membrane of the rat ankle joint (×200, scale bar is 200 μm). Red arrows indicate synovial hyperplasia and disorganized tissue architecture. Black arrows denote inflammatory cell infiltration in synovium, which are typical pathological features of MSU-induced AGA. Note: Compared with the AGA group, * *p* < 0.05, ** *p* < 0.01, there is a significant difference; ns denotes no significant difference, *n* = 6.

We modeled the rat ankle joint swollen at 4, 12, 24, and 36 h after injection of MSU suspension. The rat ankle joint circumference was measured 4, 12, 24, and 36 h after injection. At 4 and 12 h, no significant difference was observed between the SPE and AGA groups. However, at 24 and 36 h, the BC-SPE-H group showed notably lower swelling than the AGA group (*p* < 0.01). At 36 h, both BT-SPE-H and AT-SPE-L groups also exhibited markedly reduced swelling compared to the AGA group (*p* < 0.05) (Figure 2B). At 12 h (Figure 2C), the AGA group’s ankle joints were notably thicker and more swollen than the control group. In contrast, the COL group and low- and high-dose SPE in the three regions significantly reduced swelling, with BC-SPE rats showing the lowest swelling rate. The H&E staining results (Figure 2D) revealed that the synovial tissue in the normal group had a clear structure, loose spacing, neat arrangement, and no inflammatory cell infiltration. In the AGA group, synovial tissue proliferation was evident, with numerous inflammatory cells infiltrating and lymphocytes clustering, alongside noticeable fibrous tissue proliferation. In contrast, the COL and SPE administration groups showed a significant improvement in inflammatory cell infiltration and reduced fibrous tissue proliferation. These findings indicate that SPE effectively improves the histopathological morphology of the ankle synovial membrane in rats with acute gouty arthritis.

### 3.3. Effects of SPE on the Content of Inflammatory Factors in the Serum of Rats with Acute Gouty Arthritis at Different Times

**Figure 3:**
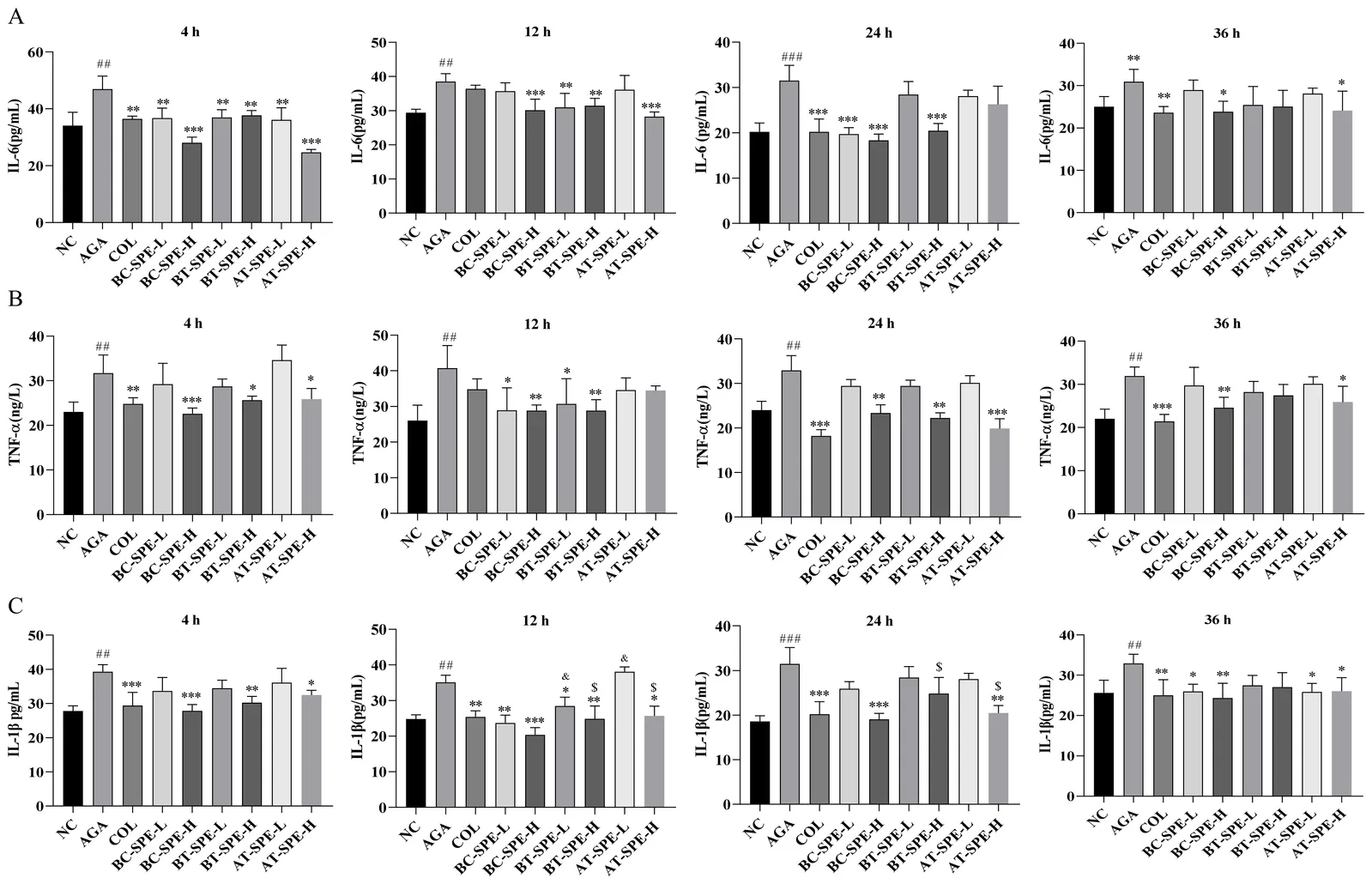
Effects of SPE on inflammatory factors IL-6, TNF-α and IL-1β in the serum of rats with AGA. (**A**) Content of IL-6 in serum. (**B**) Content of TNF-α in serum. (**C**) Content of IL-1β in serum. Compared with the NC group, ## *p* < 0.01, ### *p* < 0.001; compared with the AGA group, * *p* < 0.05, ** *p* < 0.01, *** *p* < 0.001; compared with the BC-SPE-L group, ^&^ *p* < 0.05; compared with the BC-SPE-H group, ^$^ *p* < 0.05, showing a significant difference, *n* = 6.

As shown in Figure 3, the serum levels of IL-6, TNF-α, and IL-1β in rats from the AGA group were markedly higher at 4, 12, 24, and 36 h than in the NC group (*p* < 0.01, *p* < 0.001). This suggests that the acute gout inflammation model was constructed successfully. Compared to the AGA group, BC-SPE-L notably reduced IL-6 at 4 and 24 h (*p* < 0.01, *p* < 0.001), TNF-α at 12 h (*p* < 0.05), and IL-1β at 12 and 36 h (*p* < 0.05, *p* < 0.01). BC-SPE-H significantly down-regulated these three factors at all four time points (*p* < 0.01, *p* < 0.01). BT-SPE-L reduced IL-6 (*p* < 0.01) and TNF-α and IL-1β at 12 h (*p* < 0.05). BT-SPE-H markedly decreased the levels of IL-6 and TNF-α at 4, 12, and 24 h (*p* < 0.05, *p* < 0.01, *p* < 0.001) and IL-1β at 4 and 12 h (*p* < 0.01). AT-SPE-L decreased IL-6 at 4 h (*p* < 0.01) and IL-1β at 36 h (*p* < 0.05). AT-SPE-H notably down-regulated IL-6 at 4, 12, and 36 h (*p* < 0.05, *p* < 0.001), TNF-α at 4, 24, and 36 h (*p* < 0.05, *p* < 0.001), and IL-1β at all four time points (*p* < 0.05, *p* < 0.01). At the 12 and 24 h points, BC-SPE-H produced the greatest reduction in serum IL-1β concentration among the three SPEs, with significantly lower IL-1β levels than BT-SPE-H and AT-SPE-H (*p* < 0.05).

### 3.4. Effect of SPE on HMGB-1 Protein in Rat Ankle Joint Synovial Tissue

**Figure 4:**
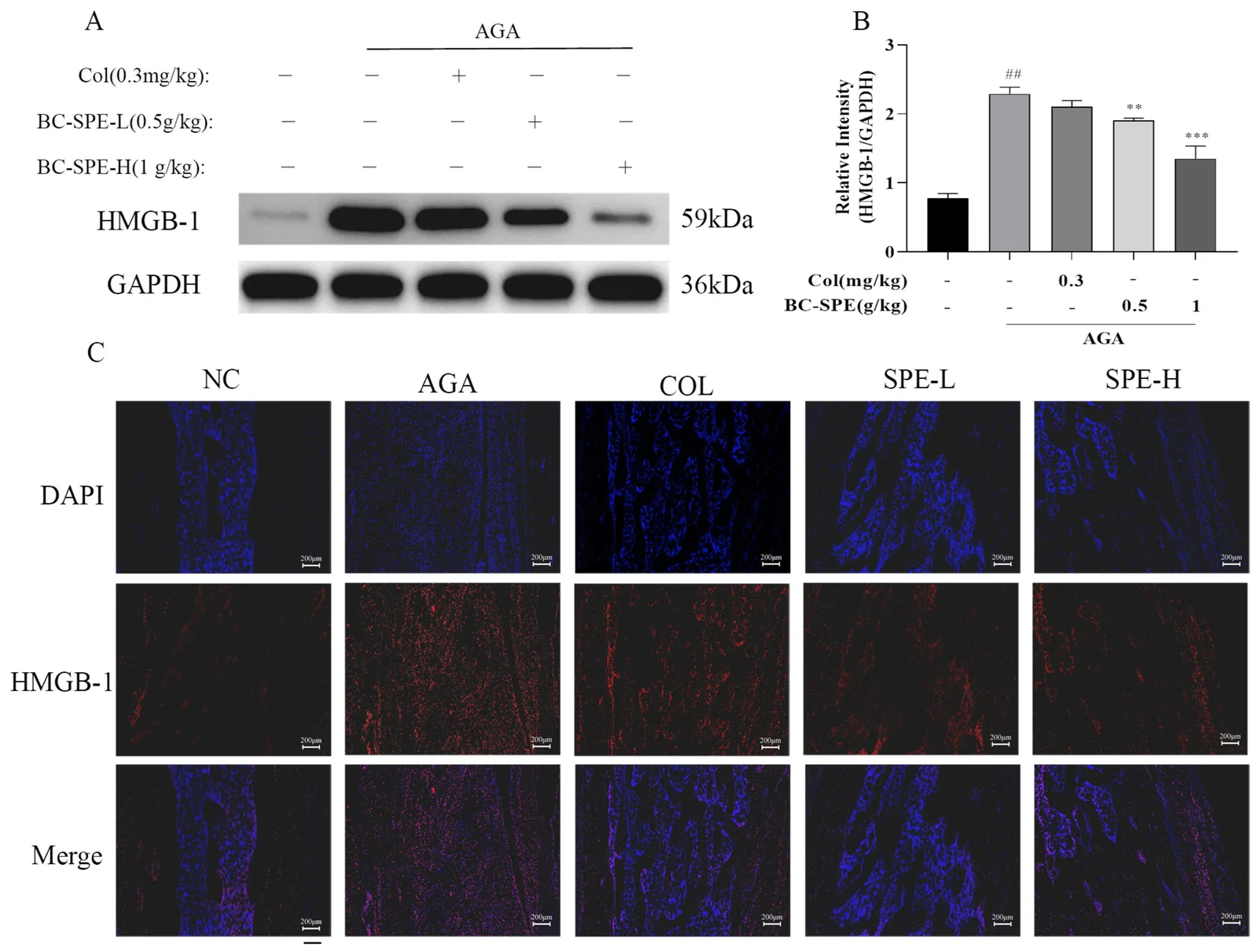
Effect of SPE on HMGB-1 protein expression in rats with AGA. (**A**,**B**) Western blotting method was used to measure joint synovial tissue HMGB-1 protein expression and optical density value statistical analysis. (**C**) Immunofluorescence analysis for measuring the ankle joint synovial tissue expression of HMGB-1. Note: Compared with the normal group, ## *p* < 0.01; compared with the AGA group, ** *p* < 0.01, *** *p* < 0.001, there is a significant difference, *n* = 6.

The swelling rates and detection results for IL-6, TNF-α, and IL-1β inflammatory factors showed that rats from the BC-SPE group maintained the lowest levels of inflammatory factors. Therefore, we focused our subsequent experiments solely on inflammation-related proteins in the ankle joint tissue of rats from the BC-SPE group. As shown in Figure 4, the protein expression level of HMGB-1 increased significantly in the AGA model compared to the NC group (*p* < 0.01). Compared with the AGA group, the expression level of HMGB-1 protein markedly decreased in the BC-SPE group (*p* < 0.01, *p* < 0.001). Immunofluorescence results also confirmed this trend and indicated that BC-SPE may exert anti-inflammatory effects by inhibiting the HMGB-1-mediated joint inflammation response pathway.

### 3.5. Effect of SPE on the Expression of TLR4/NF-κB Pathway in the Synovial Tissue of Rat Ankle Joints

**Figure 5:**
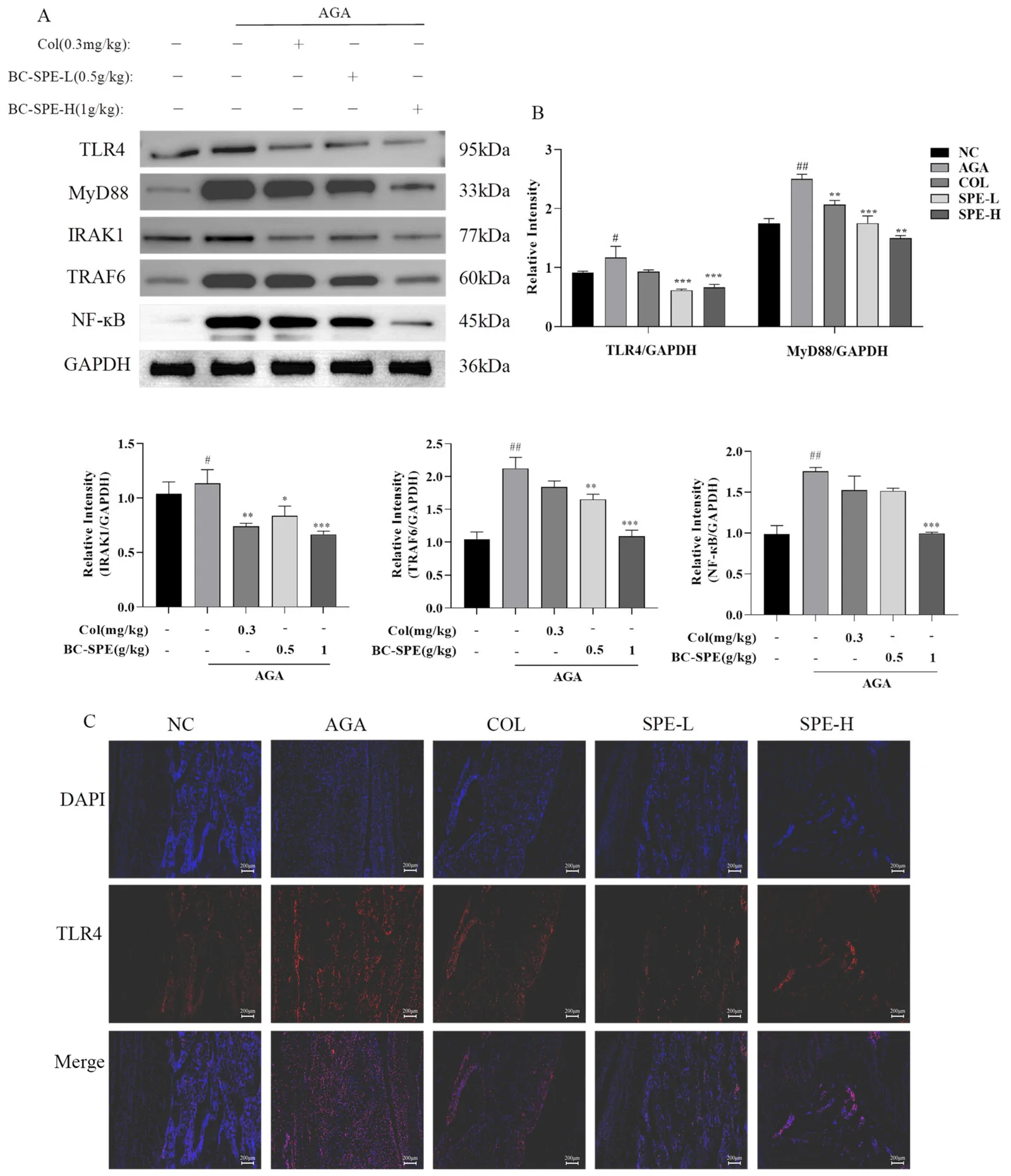
Effect of SPE on the expression of TLR4/NF-κB pathway in rats with AGA. (**A**) Expression of TLR4/NF-ĸB pathway proteins in the synovial tissue of the rat ankle joint. (**B**) Statistical analysis of optical density values of TLR4, MyD88, TRAF6, IRAK1, and NF-ĸB protein bands. (**C**) Immunofluorescence analysis to detect the expression of TLR4 in the synovial tissue of the ankle joint of AGA rats. Compared with the NC group, # *p* < 0.05, ## *p* < 0.01; compared with the AGA group, * *p* < 0.05, ** *p* < 0.01, *** *p* < 0.001, there is a significant difference, *n* = 6.

To study the mechanism by which SPE alleviates acute gouty arthritis, this study analyzed the expression levels of the TLR4/NF-ĸB signaling pathway (Figure 5). In the synovial tissue of rats in the AGA group, protein expression levels of TLR4, MyD88, IRAK1, TRAF6, and NF-ĸB were significantly elevated compared to the NC group (*p* < 0.05, *p* < 0.01). However, in the SPE group, these protein expression levels in the synovial tissue of the ankle joint were notably lower than in the AGA group (*p* < 0.05, *p* < 0.01, *p* < 0.001). Immunofluorescence detection revealed that the fluorescence intensity of TLR4 was markedly higher in the AGA group and decreased in the SPE group. These results suggest that BC-SPE may have an anti-inflammatory effect by inhibiting protein expression of the TLR4/NF-ĸB pathway and modulating local inflammation in joints.

### 3.6. Effect of SPE on the Expression of NLRP3/MAPK Pathway Proteins in the Synovial Tissue of Rat Ankle Joints

**Figure 6:**
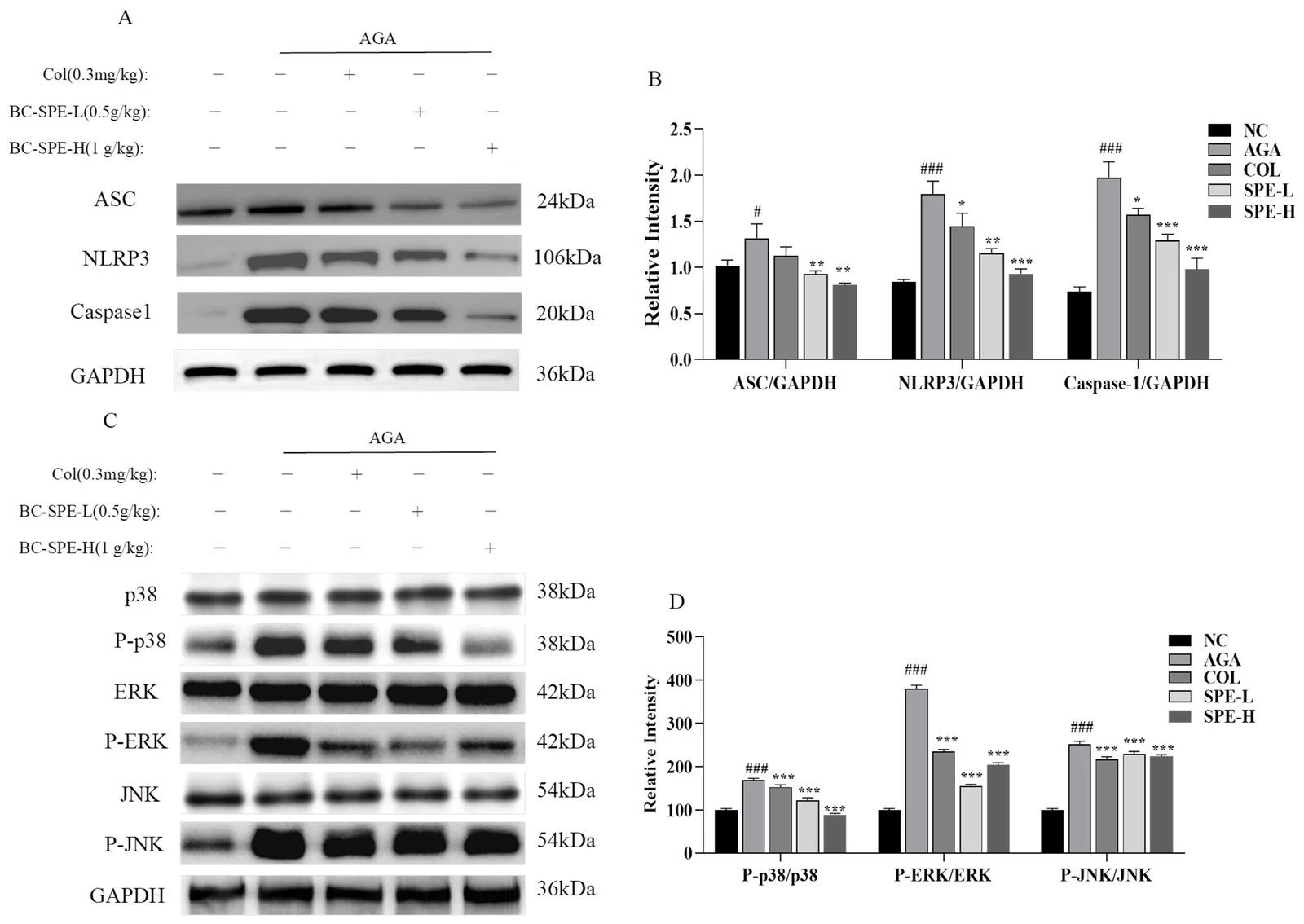
Effect of SPE on NLRP3/MAPK pathway in rats with AGA. (**A**) NLRP3 pathway protein expression in rat ankle synovial tissue. (**B**) Statistical analysis of optical density values of NLRP3, ASC, and Caspase1 protein bands. (**C**) Representative bands of MAPK pathway protein expression in rat ankle synovial tissue. (**D**) Statistical analysis of optical density values of protein bands of P-p38/p38, P-ERK/ERK, and P-JNK/JNK. Compared with the normal group, # *p* < 0.05, ### *p* < 0.001; compared with the AGA group, * *p* < 0.05, ** *p* < 0.01, *** *p* < 0.001, there is a significant difference, *n* = 6.

The NLRP3 inflammasome has been identified as a key player in acute gouty arthritis. We studied its effect on the NLRP3 inflammasome as shown in Figure 6. Compared with the NC group, Western blot analysis showed that protein expression levels of NLRP3, ASC, and Caspase1 were markedly elevated compared with the NC group (*p* < 0.05, *p* < 0.001). Additionally, the protein expression levels of P-c-Jun *N*-terminal kinase (JNK), P-extracellular signal-regulated kinase (ERK), and P-p38 mitogen-activated protein kinasep38 (p38) were markedly increased (*p* < 0.001). In contrast, the COL and SPE treatment groups exhibited a significant reduction in the protein expression levels of NLRP3, ASC, and Caspase1 (*p* < 0.05, *p* < 0.01, *p* < 0.001), as well as in P-JNK, P-ERK, and P-p38 (*p* < 0.001). These results suggest that SPE can inhibit the protein expression levels of NLRP3 and MAPK pathways, which may be anti-inflammatory in rats with acute gouty arthritis.

## 4. Discussion

Acute gouty arthritis occurs when the immune system is activated excessively by the presence of MSU crystals, which leads to many macrophages and neutrophils infiltrating with proinflammatory compounds such as IL-1, TNF, and IL-6, causing severe joint redness, swelling, heat, and pain. Core treatment includes anti-inflammation and inhibition of the activation of the inflammatory cascade [16,17,18]. In this study, an AGA model was established by injecting MSU suspension into the ankle joints of rats. After modeling, rats were characterized by a significant increase in swelling of the ankle joints, restricted movement, and significant inflammatory cell infiltration in synovial tissue. SPE intervention was able to reduce swelling of joints and synovial damage, consistent with the anti-inflammatory potential of botanical extracts observed in other crystal-induced arthropathies [9,18,19]. This shows that SPE has a clear protective effect on MSU-induced acute gouty inflammation.

Network pharmacology prediction indicated that sunflower receptacle’s anti-gout properties target proteins such as HMGB1, TLR4, TNF-α, and Caspase1, mainly enriched with TLR4, MAPK, NF-ĸB, and inflammation-related pathways. HMGB1 plays an important role in this target network; however, there are few studies on its role as a key node in acute gouty arthritis treatment that suggest significant potential for exploration. As a damage-associated protein, HMGB1 acts as a late-stage inflammatory factor outside cells, binding to TLR4 to trigger downstream inflammatory signals [20]. MSU crystals can stimulate active release of HMGB1, binding to TLR4, recruiting MyD88 to form a signaling complex that activates NF-κB and the NLRP3 inflammasome, and activating IL1β to mature and release IL1β as an axis for gout attacks [19,21]. This study demonstrated that, compared to the NC group, protein expression of HMGB1 and TLR4 was significantly higher. Compared with the AGA group, SPE notably reduced HMGB1 and TLR4 levels, suggesting that SPE has anti-inflammatory activity by inhibiting activation of the HMGB1-TLR4 axis.

MyD88 is an adapter protein of TLR4. It recruits IRAK4 and IRAK1 to form complexes, activates TRAF6, and activates both NF-ĸB and MAPK pathways [22,23]. Once TRAF6 is activated, it influences these signaling pathways. Activated NF-ĸB translocates to the nucleus, where it directly activates transcription of inflammatory factors such as IL-1β, TNF-α, and IL-6. In the MAPK pathway, ERK, JNK, and p38 activate activator protein-1 (AP-1) through a phosphorylation cascade, enhancing the inflammatory response [24,25]. Our results showed a significant increase in the levels of MyD88, IRAK1, TRAF6, and phosphorylated forms of ERK, JNK, and p38 in the AGA group. SPE was found to inhibit the expression of these molecules, indicating that it can block the activation of both NF-ĸB and MAPK pathways, thereby suppressing inflammatory transcription and amplification.

NLRP3 inflammasome assembly and Caspase-1 activation, which cleaves pro-IL-1β to produce mature IL-1β, are critical for the amplitude of the AGA inflammatory cascade [17,26]. While NLRP3 activation is well documented in MSU crystal-driven inflammation, its upstream regulation by HMGB1–TLR4–NF-κB signaling in gouty synovium has been less clearly delineated. Our data showed that SPE reduced synovial NLRP3, ASC, and Caspase-1 expression alongside decreased serum IL-1β and IL-6, aligning with recent evidence that HMGB1-TLR4 blockade attenuates inflammasome priming and assembly in macrophage models of sterile inflammation [19,21]. These results suggest that SPE mitigates inflammasome activation and the release of pro-inflammatory factors by inhibiting the HMGB1-TLR4-NLRP3 axis, thereby improving acute gouty arthritis. All results in this study were obtained from rat models, and interspecies differences in metabolism and immune responses limit the direct clinical translation of the present findings. Therefore, further clinical validation and dose optimization are required before SPE can be applied to the clinical intervention of gouty arthritis.

Although our experiments demonstrated that SPE down-regulated HMGB1-TLR4-NLRP3 signaling and alleviated MSU-induced AGA, all mechanistic conclusions remain correlative, as they are based solely on in vivo observations. This study has three main limitations. Firstly, mechanistic conclusions remain correlative, as they are based solely on in vivo pharmacological observations without in vitro causal validation. Secondly, the chemical composition of SPE was not characterized or quantified, precluding identification of the specific active constituents. Thirdly, only male rats were used, and molecular analyses were restricted to a single post-modeling time point, which may limit generalizability. Future studies will address these gaps through in vitro mechanistic validation, comprehensive phytochemical profiling, and multi-sex, multi-time-point experiments.

## 5. Conclusions

In summary, SPE alleviates MSU-induced acute gouty arthritis in rats. These protective effects are associated with reduced HMGB1 expression, down-regulated levels of TLR4, IRAK1, and TRAF6, suppressed activation of NF-ĸB and MAPK signaling pathways, decreased NLRP3 inflammasome assembly, and reduced release of pro-inflammatory factors. The current study provides preliminary in vivo evidence for the protective effect of SPE against acute gouty arthritis and offers an experimental basis for further research, development, and application of SPE in the prevention and treatment of gout.

## Data Availability

The data presented in this study are available on request from the corresponding author due to the institutional data management policy.
